# The Value of U/S to Determine Priority for Upper Gastrointestinal Endoscopy in Emergency Room

**DOI:** 10.1097/MD.0000000000002241

**Published:** 2015-12-11

**Authors:** Abd Elrazek Abd Elrazek, Hamdy Mahfouz, Khaled Abd Elazeem, Mohamed Fakhry, Emad Abd Elrazek, Mahmoud Foad, Mohamed Alboraie, Aly Ragab, Shazly Baghdady, Shymaa E Bilasy, Khaled Salama, Ramy Abdel Masseih, Mohamed Omar Amer, Sayed Hassaneen, Akshaya Srikanth Bhagavathula, Asim Ahmed Elnour, Saif K. Al Nuaimi, Abdullah Shehab

**Affiliations:** From the Division of Liver Transplantation and Data Mining Research, Department of Hepatology and GIT; Senior Researcher (Al Azhar and Aswan University, Cairo, Asuit and Aswan, Egypt (AEAH); Department of Tropical, GI and Hepatology – Al Azhar School of Medicine-Asuit Branch-Al Azhar University-Asuit, Egypt (EMAE, HMM, MF, KAE); Department of Gynecology and Obstetrics, Al Azhar School of Medicine, Asuit Branch, Al Azhar University - Asuit, Egypt (MF); Department of Internal Medicine, Al Azhar School of Medicine, Al Azhar University, Cairo, Egypt (MA); Department of General and Laparoscopic Surgery, Al Azhar School of Medicine, Cairo (AR); Chest and Respiratory Intensive Care Unit, Aswan School of Medicine, Aswan University, Aswan, Egypt (SB); Department of Biochemistry, Faculty of Pharmacy, Suez canal University, Ismailia, Egypt (SEB); Graduate Medical Student, Egypt (KS); Department of Hepatology, UCLA, USA; Research Assistant (RAE); Department of Hepatology, National Liver Institute, Menofiya University-Menofiya, Egypt (MOA); Department of Radiology, Asuit faculty of Medicine, Asuit University, Asuit, Egypt (SH); Department of Clinical Pharmacy, University of Gondar College of Medicine and Health Sciences, Gondar, Ethiopia (ASB); Departments of Clinical Pharmacology, Medicine and Cardiovascular Diseases, College of Medicine and Health Sciences (CMHS), University of Arab Emirates (AAE, SKA, AS); UAE-Emirates (AAE, SA, AS).

## Abstract

In countries endemic for liver and GIT diseases, frequent emergency department (ED) patients contribute to a disproportionate number of visits consuming substantial amount of medical resources. One of the most frequent ED visits is patients who present with hypovolemic shock, abdominal pain, or confusion with or without signs of upper gastrointestinal bleeding (UGIB). The use of conventional two-dimensional ultrasound (2D-U/S) may provide immediate and useful information on the presence of esophageal varices, gastrointestinal tumors, and other GIT abnormalities.

The current study investigated the feasibility of using (2D-U/S) to predict the source of UGIB in ED and to determine patients’ priority for UGE.

Between February 2003 and March 2013, we retrospectively reviewed the profiles of 38,551 Egyptian patients, aged 2 to 75 years old, who presented with a history of GI/liver diseases and no alcohol consumption. We assessed the value of 2D-U/S technology in predicting the source of UGIB.

Of 38,551 patients presenting to ED, 900 patients (2.3%), 534 male (59.3%) and 366 female (40.7%) developed UGIB. Analyzing results obtained from U/S examinations by data mining for emergent UGE were patients with liver cirrhosis (LC), splenomegaly, and ascites (42.6% incidence of UGIB), followed by LC and splenomegaly (14.6%), LC only (9.4%), and was only 0.5% who had no morbidity finding by 2D-U/S.

Ultrasonographic instrumentation increases the feasibility of predictive emergency medicine. The area has recently not only gained a fresh impulse, but also a new set of complex problems that needs to be addressed in the emergency medicine setting according to each priority.

## INTRODUCTION

Upper gastrointestinal bleeding (UGIB) is a common medical condition, with high patient morbidity and medical care costs that usually presents as hematemesis and/or melena.^1–5^ Two-dimensional ultrasonography (2D-U/S) is a simple, portable, and rapid technique that can play an important role in screening for gastrointestinal (GI) and hepatobiliary diseases that cause different forms of UGIB.^6^ Additionally U/S has recently been used to assess the degree of esophageal varices (EVs) in cirrhotic patients by 2D-U/S.^7,8^ Data mining programs have identified factors significantly associated with patient mortality.^9,10^ Therefore, predictive data mining is becoming an instrumental tool in identifying higher risk patients. This study used both data mining and 2D-U/S to predict UGIB in nonalcoholic patients. We observed that predictors of UGIB were liver cirrhosis (LC) – associated portal hypertension (PHT) – induced splenomegaly and ascites using data-mining analysis.

## PATIENTS AND METHODS

Al Azhar School of Medicine, Asuit Branch, Al Azhar University, Egypt, ethics committee specifically has approved this study.

Participants provided their written informed consent to participate in this study. The participant consented form was recorded and kept with study documents. The ethics committee approved the consent procedure.

Of the 38,551 patients evaluated in the emergency room, outpatient clinic and specialized centers, or admitted to the hospital with manifestations of GI/liver disorders between February 2003 and March 2013, 900 (2.3%) nonalcoholic patients had developed UGIB (hematemesis and/or melena). Of those 900 patients, 534 (59.3%) were males and 366 (40.7%) were females, ranging in age from 2 to 75 years. All had been treated for UGIB, including hematemesis and/or melena, at the GI Centers of Al Azhar University Hospitals in Egypt. Patients were diagnosed with UGIB by upper gastrointestinal endoscopy (UGE) from February 2003 until March 2013. The demographic and clinical characteristics of these patients were analyzed to detect factors predictive of UGIB.

All patients with UGIB were evaluated by UGE and 2D-U/S. Patients were categorized by age into those <20, 21 to 30, 31 to 40, 41 to 50, 51 to 60, and >60 years.

### Statistical Analyses

All statistical analyses were performed using SPSS version 18 software for Microsoft Windows (Statistical Package for the Social Sciences; SPSS Inc., Chicago, IL).

### Data-Mining Analysis

Data-mining analysis is a process by which a computer examines large amounts of data to create an algorithm. Both naive Baÿes (10-fold cross-validation) and a decision-tree model were used. The descriptive Rapid I models of Rapid miner Program (Germany) were initially generated to determine the most significant independent variable in each stage of predicting dependent variables using the computational analysis instead of mathematical analysis.

## RESULTS

**FIGURE 1:**
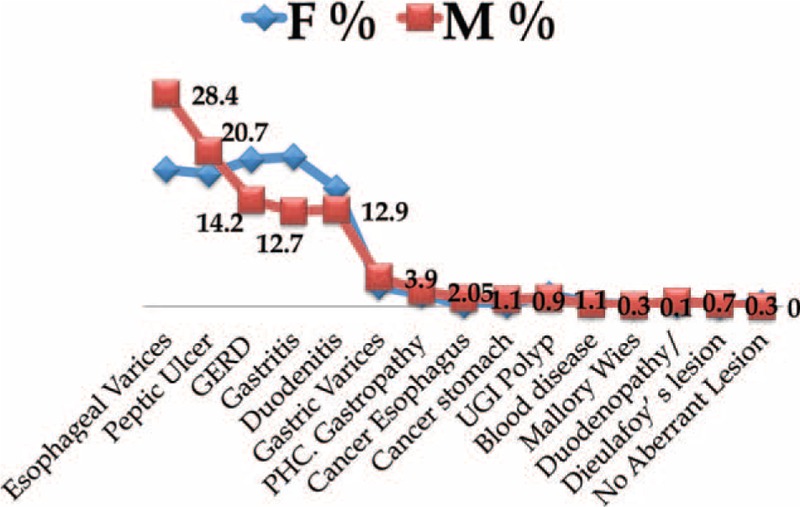
Rates of UGIB-related diseases in males and females. M = male, F = female, UGIB = upper gastrointestinal bleeding.

Of 38,551 patients aged 2 to 75 years (mean age 43.5 ± 1.2 years) and presenting with GI/liver diseases, only 900 (2.3%) had UGIB. These 900 patients included 534 males (59.3%) and 366 females (40.7%); of these, 78.1% and 87%, respectively, presented with hematemesis and 21.9% and 13%, respectively, presented with melena. The most frequent predisposing factors for UGIB included peptic ulcer, gastroesophageal reflux disease (GERD), gastritis, and duodenitis (65.8%), with PHT-associated lesions, EVs, gastric varices, portal hypertensive congestive gastropathy (PHCG), and duodenopathy accounting for 30.1% (Table 1). The frequencies of most UGIB diseases were similar in males and females, but EVs were more common in males (28.4% vs 18.3%) and GERD (19.6% vs 14.2%) and gastritis (20% vs 12.7%) were more common in females (Figure 1).

**TABLE 1:**
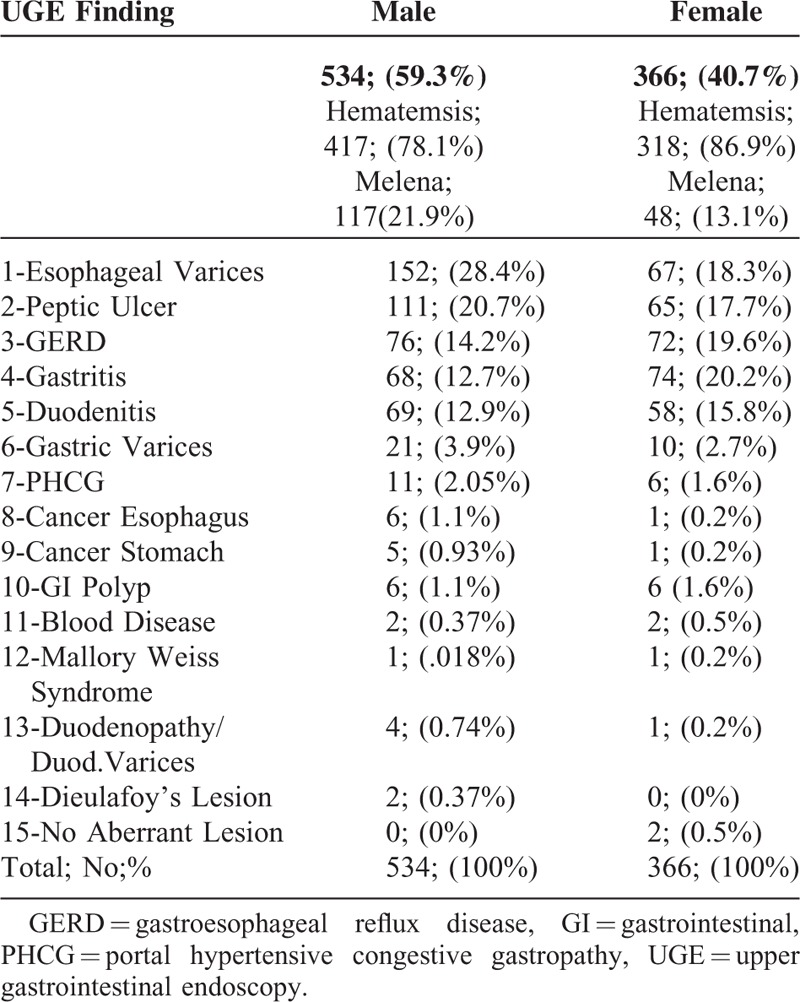
UGE Finding in Both Male and Female Groups

Clinical presentation of patients with UGIB may help in predicting diagnosis. For example, abdominal pain, heartburn, and vomiting commonly occurring in first 3 decades-related bleeding diseases. Additionally, sonographic images will help in such demonstration prediction in various age-groups, LC, diffuse hepatic pathology, splenomegaly, hepatic focal lesion, and ascites commonly occurring in 4th and 5th decades-related bleeding morbidities (Table 2).

**TABLE 2:**
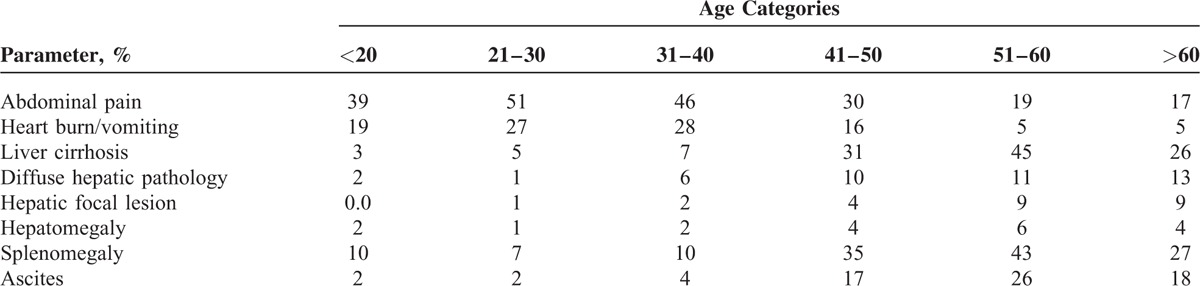
Age Categorization Clinical Presentation With Ultrasonography (U/S) Finding Investigation

**FIGURE 2:**
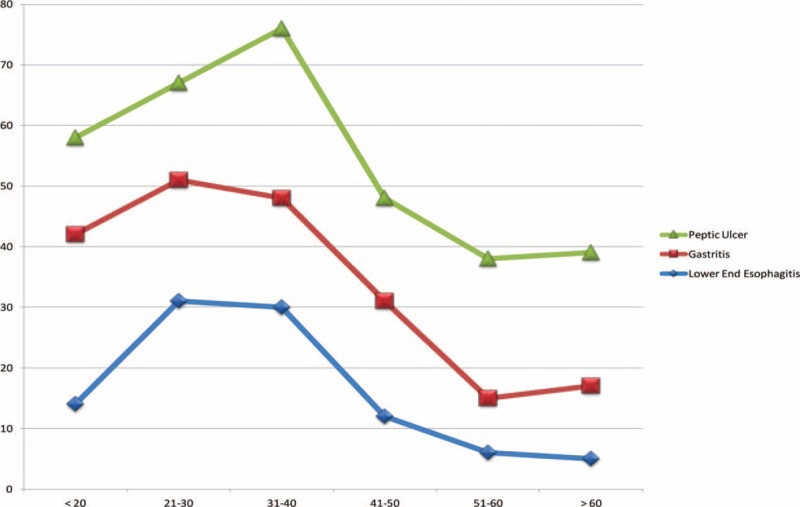
Incidence of ultrasound-detected peptic ulcer, gastritis, and lower end esophagitis in patients assorted by age groups.

**FIGURE 3:**
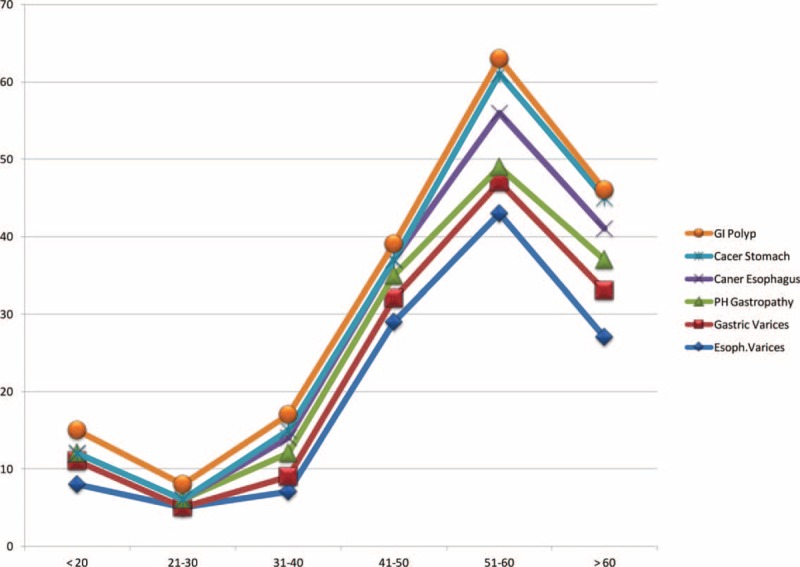
Incidence of ultrasound detected gastrointestinal polyps, stomach cancer, esophageal cancer, portal hypertensive gastropathy, gastric varices, and esophageal varices in patients assorted by age groups.

**FIGURE 4:**
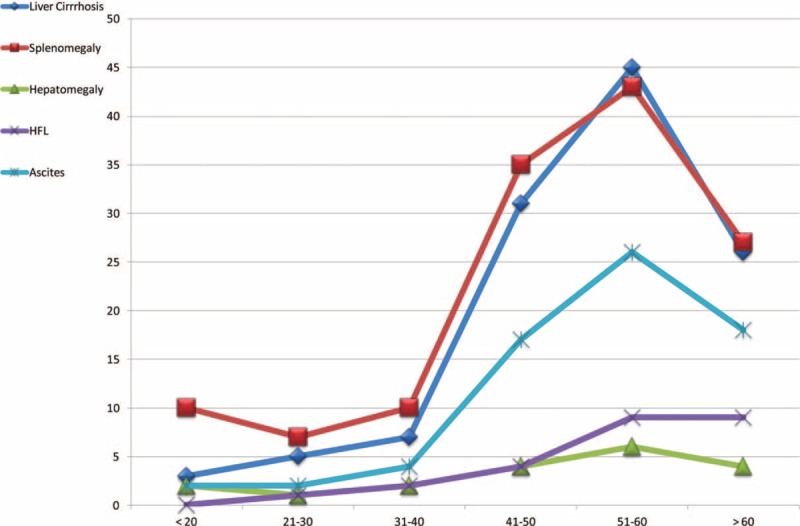
Incidence of ultrasound-detected liver cirrhosis, splenomegaly, hepatomegaly, hepatic focal lesion (HFL), and ascites in patients assorted by age groups.

Common but not serious causes of UGIB included peptic ulcer (both gastric and duodenal), gastritis, and lower end esophagitis, which occurred more frequently during the 2nd and 3rd than during other decades of life (Figure 2). Serious UGIB, with higher morbidity and mortality rates, included EVs, gastric varices, esophageal cancer, gastric cancer, PHCG, and GI polyps, these occurred more frequently during the 3rd, 4th, and 5th decades of life than in other decades (Figure 3). Sonographic images frequently supported the diagnoses of serious UGIB (Figure 4).

**FIGURE 5:**
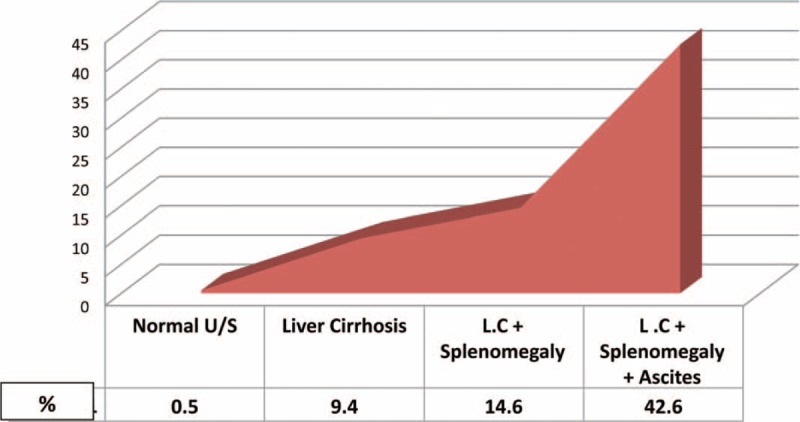
Prediction of upper gastrointestinal bleeding (UGIB) according to ability of conventional trans-abdominal 2D ultrasound to predict UGIBs.

**FIGURE 6:**
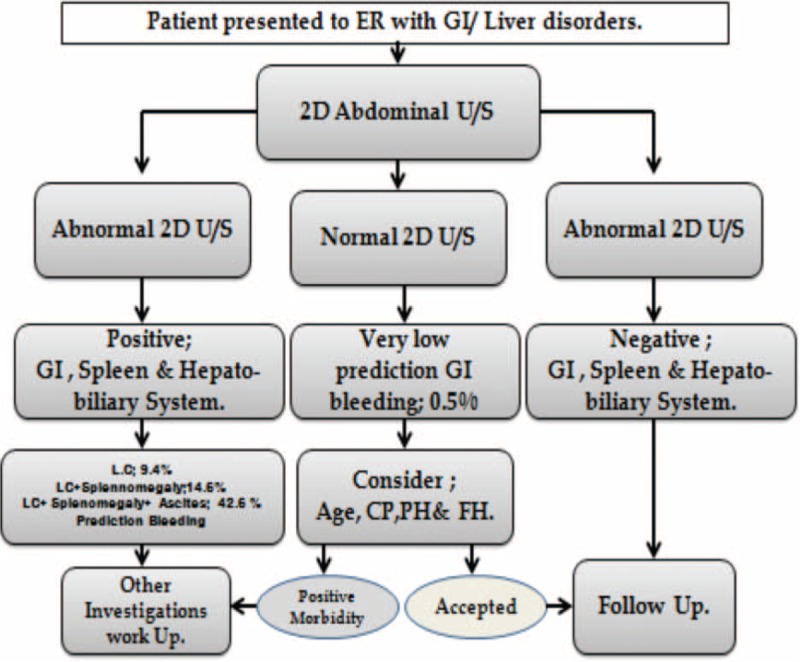
Modified flow chart algorithm of sequential steps used by Rapid I, ver.4.6, to predict upper gastrointestinal bleeding (UGIB).

The decision tree algorithm of Rapid I ver. 4.6 Berlin, was able to predict UGIB 42.6% of patients with LC, splenomegaly, and ascites, but in only 14.6% of patients with LC-associated PHT or splenomegaly and in only 9.4% of patients with LC alone. Only 0.5% of patients negative for morbidity by 2D-U/S showed signs of bleeding (Figure 5). However, it was 14.6% in LC-associated PHT splenomegaly and only 9.4% in those presented with only LC. Those without any morbidity finding by 2D-U/S technology showed 0.5% incidence bleeding (Figure 5). A modified algorithm was developed to enable 2D-U/S to predict UGIB in patients presenting with GI and/or hepatic disorders (Figure 6).

## DISCUSSIONS

UGIB is a common medical emergency, estimated to affect 150 per 100,000 adults per year and with inpatient mortality rate as high as 10%. The most common causes of UGIB include gastric and/or duodenal ulcers, esophagogastric varices with or without PHCG, gastric antral vascular ectasia (GAVE), esophagitis, erosive gastritis/duodenitis, Mallory–Weiss syndrome, angiodysplasia, mass lesions (polyps/cancers), and Dieulafoy lesion. Less frequent causes of UGIB include hemophilia, hemosuccus pancreatitis, and aortoenteric fistula.^11^ Given the high prevalence of viral hepatitis in Egypt, the usual load of emergency rooms and the urgent care that UGIB patients need, therefore, identifying high risk UGIB patients by data mining will save much need time, effort and will ultimately improve the patient care.

This study used data-mining analysis to retrospectively examine the demographic and clinical characteristics of 900-UGIB patients, thereby enabling factors leading to the development of these conditions and their sequelae to be identified. Preexisting liver disease associated PHT was the most prominent predisposing factor for UGIB, being present in 30.1% of patients. However, peptic ulcer, GERD, gastritis, and duodenitis accounted were observed in 65.8% of patients with UGIB.

The initial evaluation of a patient with a suspected acute UGIB includes brief personal and family history, physical examination, laboratory tests, U/S investigation, and for some patients, nasogastric lavage. This evaluation is performed to assess the severity of the bleeding, identify potential bleeding sources, and determine if any preexisting conditions that may affect subsequent management. UGE remains the gold standard for the diagnosis of esophago-gastroduodenal lesions. In contrast, other noninvasive biochemical and radiologic parameters have been inaccurately reported as capable of replacing endoscopic screening.^12–14^ However, U/S can save time, money, and enhance the ability to diagnose/predict the underlying cause of UGIB. Moreover, conventional abdominal U/S can detect many liver diseases and hepatic focal lesions, and provide useful data for the presence of PHT and EVs in cirrhotic patients. 2D-U/S has been used to detect risky EVs in cirrhotic patients.^15^ Initial evaluations in patients with suspected UGIB are used to guide decisions regarding triage, resuscitation, empiric medical therapy, and diagnostic testing.

The overall prevalence of UGIB in the current study was higher (2.3% over 10 years in patients with GI/liver diseases) than the rates reported in other countries. For example, a study from one large health maintenance organization in the United States found that the annual incidence of hospitalization for acute UGIB was approximately 100 per 100,000 adults, was twice as frequent in males as in females and increased with age.^16^ High UGIB rates due to variceal factors in endemic areas as Egypt is likely due to the incidence and severity of chronic viral hepatitis.

Viral hepatitis appears to affect males more than females, and its complications are more pronounced in males than in females. Indeed, we observed a higher prevalence of EVs in males than in females (28.4% vs 18.3%). Farming-related water activities are among the primary occupations of men living in rural areas across the Nile delta and coinfected with viral hepatitis and schistosomiasis. The percentage of males exposed to schistosomal infection and parenteral antischistosomiasis therapy is increasing. Other factors increasing the rates of UGIB, including higher rates for unsafe medical practices related to HCV and HBV transmission as needle injections among males. Alternatively, hormonal factors may enable females to more readily clear viral hepatitis, especially in patients infected with acute stage HCV, to the extent that estrogen was previously reported to inhibit the production of HCV infectious particles in cell culture systems. The presence of certain single nucleotide polymorphisms in the gene-encoding estrogen receptor α can enable females to spontaneously clear persistent infection by HCV. In addition, estrogen inhibits the production of reactive oxygen species, the activation of hepatic stellate cells, and early hepatocyte apoptosis. This may delay the progression of hepatic fibrosis in females.^17^ However, the higher prevalence of UGIB-related GERD and gastritis in females may be associated with the higher worldwide prevalence of obesity in females than in males. For example, in Egypt, 26.4% of men and 48.4% of women were found to be obese.^18–30^ Obesity is a risk factor associated with GERD and other GI disorders.^31–32^

Evaluation of age-associated UGIB may improve early diagnosis and further management. Serious UGIB commonly occurs during the 4th, 5th, and 6th decades of life, together with related sonographic images, whereas nonserious UGIB, with lower mortality and morbidity rates, occurs more frequently during the 2nd and 3rd decades of life. GI polyps should be histopathologically evaluated in all age groups, since they may be cancerous or precancerous in patients with hematemesis and a family history of GI tumors.

Predictive data mining is becoming an essential instrument in medicine, including in the evaluation of morbidity and mortality in patients with GI and liver diseases. Understanding the main issues underlying these methods and the application of agreed and standardized procedures is mandatory for their deployment and evaluation of UGIB-related morbidities.

In this study, the decision-tree algorithm showed a high-incidence association of UGIB with the occurrence of LC, splenomegaly, and ascites, 42.6%. It was 14.6% in LC and splenomegaly (compensated group sonographically), 9.4% in those with LC only. However, only 0.5% of patients developed UGIB if there is a negative sonographic picture-related GI and hepatobiliary systems (Figure 5).

## CONCLUSIONS

Ultrasonography should be a diagnostic urgent investigation in the emergency room and this will increase the ability to predict UGIB in patients with GI and liver diseases with special concern for countries that are endemic with LC-related morbidities.

## LIMITATIONS OF THE STUDY

This study was performed in Egypt, which has one of the highest rates of endemic HCV infection worldwide. Thus, results in countries with lower rates of HCV infection may differ markedly. Therefore, our results may require confirmation in other countries and in other diverse ethnic groups.
