# The Value of U/S to Determine Priority for Upper Gastrointestinal Endoscopy in Emergency Room: Erratum

**DOI:** 10.1097/01.md.0000479433.86506.88

**Published:** 2016-01-08

In the article “The Value of U/S to Determine Priority for Upper Gastrointestinal Endoscopy in Emergency Room”,^1^ which appeared in Volume 94, Issue 49 of *Medicine*, several typos appeared in the “Limitations of the Study” section. Also, the author's degrees were not given in full in the byline. The article has since been corrected online.
